# Strain-Specific Probiotic Properties of Bifidobacteria and Lactobacilli for the Prevention of Diarrhea Caused by Rotavirus in a Preclinical Model

**DOI:** 10.3390/nu12020498

**Published:** 2020-02-15

**Authors:** Ignasi Azagra-Boronat, Malén Massot-Cladera, Karen Knipping, Johan Garssen, Kaouther Ben Amor, Jan Knol, Àngels Franch, Margarida Castell, María J. Rodríguez-Lagunas, Francisco J. Pérez-Cano

**Affiliations:** 1 Physiology Section, Department of Biochemistry and Physiology, Faculty of Pharmacy and Food Science, University of Barcelona (UB), 08028 Barcelona, Spain; ignasiazagra@ub.edu (I.A.-B.); malen.massot@ub.edu (M.M.-C.); angelsfranch@ub.edu (À.F.); margaridacastell@ub.edu (M.C.); franciscoperez@ub.edu (F.J.P.-C.); 2 Nutrition and Food Safety Research Institute (INSA-UB), 08921 Santa Coloma de Gramenet, Spain; 3 Danone Nutricia Research, 3584 CT Utrecht, The Netherlands; karen.knipping@danone.com (K.K.); johan.garssen@danone.com (J.G.); kaouther.benamor@danone.com (K.B.A.); jan.knol@danone.com (J.K.); 4 Division of Pharmacology, Utrecht Institute for Pharmaceutical Sciences, Faculty of Science, Utrecht University, 3584 CA Utrecht, The Netherlands; 5 Laboratory of Microbiology, Wageningen University, 6708 WE Wageningen, The Netherlands

**Keywords:** probiotic, rotavirus, diarrhea, suckling rats

## Abstract

Probiotic supplementation with different lactobacilli and bifidobacterial strains has demonstrated beneficial effects in infectious diarrhea caused by rotavirus (RV) in young children. Preclinical models of RV infection might be a good strategy to screen for the efficacy of new probiotic strains or to test their comparative efficacy. Neonatal Lewis rats were supplemented with *Bifidobacterium breve* M-16V, *Lactobacillus acidophilus* NCFM, *Lactobacillus helveticus R0052*, or *Lactobacillus salivarius* PS2 from days 2–14 of life. On day five, animals received RV SA-11 orally. Fecal samples were collected daily, weighed, and scored for the calculation of severity and incidence of diarrhea. In addition, fecal pH and fecal viral shedding were measured. Animals were sacrificed at the end of the study and their blood was obtained for the quantification of RV-specific immunoglobulins. RV infection was induced in ~90% of the animals. All probiotics caused a reduction of several clinical variables of severity and incidence of diarrhea, except *L. salivarius* PS2. *L. acidophilus* NCFM, *B. breve* M-16V, and *L. helveticus* R0052 seemed to be very effective probiotic strains. In addition, all *Lactobacillus* strains reduced the viral elimination one day post-inoculation. No differences were detected in the specific anti-RV humoral response. The present study highlights the strain-specific effects of probiotics and identifies promising probiotics for use in ameliorating and preventing RV-induced diarrhea in children, for example by including them in infant formulas.

## 1. Introduction

Probiotics are defined as “live microorganisms that, when administered in adequate amounts, confer a health benefit on the host” [1]. Several commensal microorganisms with probiotic properties are known to reside in the gastrointestinal tract of humans as part of the microbiota [2]. In this context, probiotics are currently being used to prevent illness, improve health, and treat intestinal diseases such as infections triggered by numerous pathogens, including viruses [2,3,4]. In the past decades there has been an increasing interest in the use of probiotics to prevent and ameliorate viral diarrhea [5,6].

The most common viral forms of gastroenteritis are caused by the rotavirus (RV), norovirus, adenovirus, and astrovirus [7,8]. RV is the main cause of diarrheal death among children <5 years worldwide, accounting for 146,000 deaths in 2015 [9]. The disease burden of RV is substantial in both developed and developing countries; in fact, almost every child has been infected by the age of 5, and probably more than once, because reinfections are frequent [10,11,12]. Although RV vaccines are available to prevent the infection, they are not globally implemented due to their high cost, cold storage requirements, and the lower protection they offer in developing countries as a consequence of malnutrition, micronutrient deficiencies, and suboptimal breastfeeding [13,14]. Treatment of RV diarrhea mainly relies on oral rehydration solutions to replace fluid loss. However, this strategy is not effective in reducing the severity and shortening the duration of the disease [15,16].

Several studies have reported beneficial outcomes in terms of RV severity and duration after the use of probiotics. Among the mechanisms involved in the protection or amelioration of RV infection, it has been reported that probiotics may reinforce the mucosal barrier [5,17], stimulate the production of antimicrobial substances (such as lactic acid or H_2_O_2_) [7,18,19] and mucins [20,21], modulate the innate and adaptive immune response [5,22,23,24], interfere in the adherence and replication process [25,26,27], and reduce chloride secretion and oxidative stress [28].

Prevention and management of RV infection and symptoms in babies have been mainly studied using *Lactobacillus* and *Bifidobacterium* strains [5,7,29,30,31]. However, before moving to clinical trials, the safety and efficacy of probiotics need to be proven in preclinical models. In this sense, preclinical evidence has been accumulated for the following species: *Lactobacillus casei* [32,33,34], *Lactobacillus plantarum* [31], *Lactobacillus rhamnosus* GG [5,14,26,35], *Bifidobacterium adolescentis* [34], *Bifidobacterium bifidum* [29], *Bifidobacterium infantis* [29], *Bifidobacterium breve* [13,36], and *Bifidobacterium lactis* [35]. In addition, these previous studies led to the further study of the clinical evidence for the following species: *Lactobacillus acidophilus* [16,20,32,37], *L. casei* [26], *Lactobacillus reuteri* [38,39], *L. rhamnosus* GG [16,17,20,22,40,41,42,43], *Lactobacillus sporogenes* [44,45], *B. lactis* [46,47,48], and *Bifidobacterium longum* [16,20,37].

Some studies show the efficacy of certain probiotics for preventing and ameliorating RV diarrhea, but little is known about the strain-specific compared efficacy. Therefore, the present study aimed to establish and compare the strain-specific activity of four probiotics belonging to different *Bifidobacterium* and *Lactobacillus* genera and origin sources (feces, dairy culture, and breastmilk), with potential for pediatric use, in RV diarrhea induced in neonatal rats. 

## 2. Materials and Methods 

### 2.1. Animals

G15 pregnant Lewis rats (LEW/OrlRj, *n* = 18) were obtained from Janvier Labs (Le Genest-saint-Isle, France), and for the purpose of nesting and undisturbed delivery individually housed in cages (2184L Eurostandard Type II L, Tecniplast, West Chester, PA, USA) containing bedding of large fibrous particles (Souralit 1035, Bobadeb S.L., Santo Domingo de la Calzada, Spain) and tissue papers (Gomà-Camps S.A.U., La Riba, Spain). Pregnant rats were monitored daily and allowed to deliver at term. The day of birth was established as day 1 of life. On day 2, litters were randomly assigned to the six experimental groups (three dams with their litters/group) and culled to eight pups per lactating dam, with a similar number of each sex in each litter. Dams had free access to a commercial diet corresponding to the American Institute of Nutrition 93M formulation [49] (Teklad Global Diet 2014, Envigo, Indianapolis, IN, USA) and water. To avoid the influence and disturbance of biological rhythms, animal handling was performed on a scheduled basis during the first hours of the light phase. Daily handling and oral administration were performed after separating all the mothers and keeping the pups in the home-cage. Afterwards, the dam was reunited with the whole litter. Animals were housed under controlled conditions of temperature and humidity in a 12-h light/12-h dark cycle, in the Faculty of Pharmacy and Food Science animal facility (University of Barcelona, Spain). All experimental procedures were conducted in accordance with the institutional guidelines for the care and use of laboratory animals and were approved by the Ethical Committee for Animal Experimentation of the University of Barcelona and the Catalan Government (CEEA-UB Ref. 74/05 and DAAM 3046, respectively), in full compliance with national legislation following the EU-Directive 2010/63/EU for the protection of animals used for scientific purposes.

### 2.2. Experimental Design

Upon natural delivery, newborn rats were distributed into six groups of 24 animals each (three litters of eight animals/group): the reference (REF) group, the rotavirus-infected (RV) group, and four rotavirus-infected groups supplemented with: (1) *Bifidobacterium breve* M-16V, isolated from feces of a healthy infant [50]; (2) *Lactobacillus acidophilus* NCFM, isolated from adult human feces [51]; (3) *Lactobacillus helveticus R0052,* isolated from dairy culture [52]; and (4) *Lactobacillus salivarius* LMG P-27027 also referred to as *Lactobacillus salivarius* PS2, isolated from human milk [53]. All supplementations were provided by Danone Nutricia Research (Utrecht, The Netherlands).

Suckling rats received oral administration once daily, as previously described [13], with the same normalized volume/body weight of vehicle or probiotics (5.5 µL/g/day), from days 2–14 of life, corresponding to the strict lactation period. The probiotics were administered at a dose of 1 × 10^9^ CFU/100 g/day. Finally, the REF and RV groups received the same volume of water.

The RV (simian SA-11) was obtained as previously described [54], and inoculation took place at day 5 of life (4 × 10^8^ Tissue Culture Infectious Dose 50 (TCID50)/rat) in all the experimental groups with the exception of the REF group, which received the same volume of phosphate-buffered solution (PBS) under the same conditions. 

Body weight was recorded daily, and the weight gain was studied in the pre-diarrhea, diarrhea, and post-diarrhea periods. Moreover, the body mass index (BMI) was calculated as body *weight/length^2^* (g/cm^2^), and the Lee Index was calculated as (*weight*^0.33^/*length*) × 1000 (g^0.33^/cm). Animals were sacrificed on day 14 in order to analyze the effects of the supplementations once the diarrhea was resolved. 

### 2.3. Sample Collection and Clinical Indices

Fecal sampling was performed once daily throughout the study (from day 4 to day 14 of life) by gently pressing and massaging the abdomen. Fecal samples were stored at −20 °C for the analysis of RV shedding and pH measurement. Severity of diarrhea was expressed by fecal weight and by scoring fecal samples from 1 to 4 (diarrhea index, DI) based on color, texture, and amount, as follows: normal feces (1); soft yellow-green feces (2); totally loose yellow-green feces (3); high amount of watery feces (4). Scores ≥2 indicate diarrheic feces, whereas scores <2 indicate absence of diarrhea [54]. The severity-area under the curve (S-AUC) during days 5–11, coinciding with the period in which animals displayed diarrhea, was calculated as a global value of severity. The maximum severity (MS) was defined as the highest score during the period with diarrhea.

Incidence of diarrhea was expressed by the percentage of diarrheic animals (%DA), which was based on the percentage of animals displaying scores of DI ≥ 2 in each group. The incidence-area under the curve (I-AUC) during days 5–11, coinciding with the period in which animals displayed diarrhea, was calculated as a global value of incidence. The maximum incidence (MI) was defined as the highest %DA during the diarrhea period. 

The beginning and final day of diarrhea (BDD and FDD, respectively) were recorded. The diarrhea period (DP) was calculated as the interval between BDD and FDD. The days with diarrhea (DwD) were calculated by counting the actual number of days in which the animals displayed DI ≥ 2 within the DP.

At days 8 and 14, half of each litter were intramuscularly injected with ketamine (90 mg/kg) (Merial Laboratories S.A., Barcelona, Spain) and xylazine (10 mg/kg) (Bayer A.G., Leverkusen, Germany) to induce terminal anesthesia. Plasma was obtained after blood centrifugation and kept at −20 °C for immunoglobulin (Ig) analysis.

### 2.4. Fecal SA11 Shedding

Fecal samples from day 6 (1 day post-inoculation, DPI) were diluted in PBS (10 mg/mL) and homogenized using Pellet Pestles Cordless Motor (Sigma-Aldrich, Madrid, Spain). Homogenates were centrifuged (170 *g*, 5 min, 4 °C) and supernatants were frozen at −20 °C until analysis. SA-11 virus particles were quantified by ELISA, as previously described [55]. Briefly, wells were coated with anti-p42 Mab (Meridian Life Science, Memphis, TN, USA) and blocked with PBS–BSA 1%. Afterwards, samples were added to PBS-Tween–1% BSA (3 h, room temperature (RT)). Polyclonal sheep anti-RV peroxidase-conjugated antibody (MyBioSource, San Diego, CA, USA) was added (2 h, RT). Captured SA-11 virus particles were quantified by adding substrate solution and measuring the absorbance on a microtiter plate photometer (Labsystems, Helsinki, Finland) after stopping the enzymatic reaction with 3 M H_2_SO_4_. Data were interpolated by means of Multiskan Ascent v.2.6. software (Thermo Fisher Scientific SLU, Barcelona, Spain). Titrated dilutions of inactivated SA-11 particles, ranging from 10^6^ to 10^4^ / mL, were used as standard curve.

### 2.5. Fecal pH Measurement

The fecal pH was determined by means of a surface electrode coupled to a pH-meter (Crison 52 07, L’Hospitalet de Llobregat, Spain). The fecal sample was diluted with distilled water up to a concentration of 200 mg/mL and placed in a petri dish. The measures were performed in triplicate for each sample.

### 2.6. Specific Humoral Response

Plasma concentrations of anti-RV total Ig and IgM on day 14 were quantified by ELISA technique. Hence, 96-well plates (Nunc Maxisorp, Wiesbaden, Germany) were coated with UV-inactivated SA-11 at 10^5^ particles/mL. Then, plates were blocked with PBS–1% bovine serum albumin and diluted sera (1/8) was added. After washing, polyclonal anti-rat Ig-peroxidase for the quantification of total Ig (Dakocytomation, Glostrup, Denmark) or biotin anti-rat IgM G53-238 Mab (BD Biosciences, Heidelberg, Germany) was added. Subsequently, peroxidase-conjugated extravidin (Sigma-Aldrich, Madrid, Spain) was added. Finally, the substrate solution of o-phenylenediamine and hydrogen peroxide was added. After stopping the enzymatic reaction with 3 M H_2_SO_4_, the absorbance was measured at 492 nm using a microtiter plate photometer (Labsystems, Helsinki, Finland). Data were interpolated by means of Multiskan Ascent v2.6 software (Thermo Fisher Scientific SLU, Barcelona, Spain). The standard used was pooled sera of RV-inoculated dams ranging from 1/80 to 1/5120 for the analysis of total Ig and from 1/8 to 1/800 for the analysis of IgM. The highest dilution in each analysis corresponded to 1 arbitrary unit (AU).

### 2.7. Statistical Analysis

The Statistical Package for the Social Sciences (SPSS v22.0) (IBM, Chicago, IL, USA) was used for statistical analysis. Data were tested for homogeneity of variance and normality distribution by the Levene’s and Shapiro–Wilk tests, respectively. When data was homogeneous and had a normal behavior, conventional one-way ANOVA test followed by the post hoc Bonferroni was performed. Otherwise, the nonparametric Kruskal–Wallis test followed by the post hoc Mann–Whitney U (MWU) test was performed. Finally, the chi-square test was used to compare percentages of diarrhea incidence. Significant differences were established when *p* < 0.05.

The number of pups in each group was established by the Appraising Project Office’s program from the Universidad Miguel Hernández de Elche (Alicante), which allowed the detection of statistically significant differences among groups assuming that there was no dropout rate and a type I error of 0.05 (two-sided). Moreover, independently of the number of animals obtained before, at least three litters were required for each group, as previous studies demonstrated remarkable variability between litters [55]. According to the estimation performed, three litters of eight animals per group were sufficient. The final number of animals was not affected by the dropouts or outliers, as they did not occur in the present study.

## 3. Results

### 3.1. Growth and Morphometry

**Figure 1:**
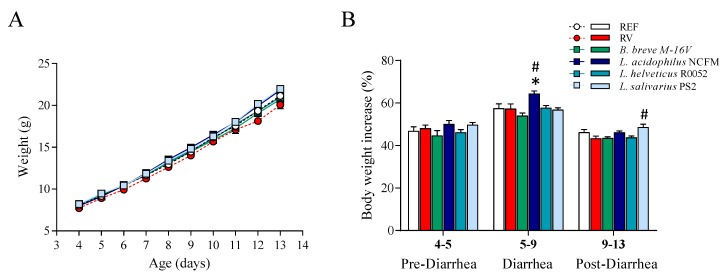
Assessment of animal growth. (**A**) Graph displaying the mean animal weight, which was measured daily from days 4 to 13 of life. (**B**) Graph displaying the body weight increase in the pre-diarrhea, diarrhea and post-diarrhea periods. Results are expressed as mean ± S.E.M. (*n* = 24/group). * *p* < 0.05 compared to the reference (REF) group; ^#^ *p* < 0.05 compared to the rotavirus-infected (RV) group. *B*.: *Bifidobacterium*; *L*.: *Lactobacillus*.

The weight of the animals was similar in most of the groups throughout the study (Figure 1A). Moreover, RV infection did not induce body weight loss, as shown on comparison to the REF group. However, although some differences appeared in specific days due to the probiotic supplementations, only the group receiving *L. salivarius* PS2 showed consistently higher body weight throughout the study compared to the RV group (*p* < 0.05). In terms of body weight gain (Figure 1B), the *L. acidophilus* NCFM group displayed ~7% more weight gain in the diarrhea period and the *L. salivarius* PS2 group ~5% more in the post-diarrhea period, as compared to the RV group.

The analysis of morphometric variables was performed at the end of the study (Table 1). Although we did not observe body weight loss due to RV infection, the body mass index of the RV group was slightly lower than that of the REF group (*p* < 0.05). In addition, the supplementation with *L. salivarius* PS2 induced higher BMI and Lee Index (*p* < 0.05), confirming the previous results on body weight. The rest of the supplementations did not show any effects on these variables.

### 3.2. Clinical Evaluation of Diarrhea

**Figure 2:**
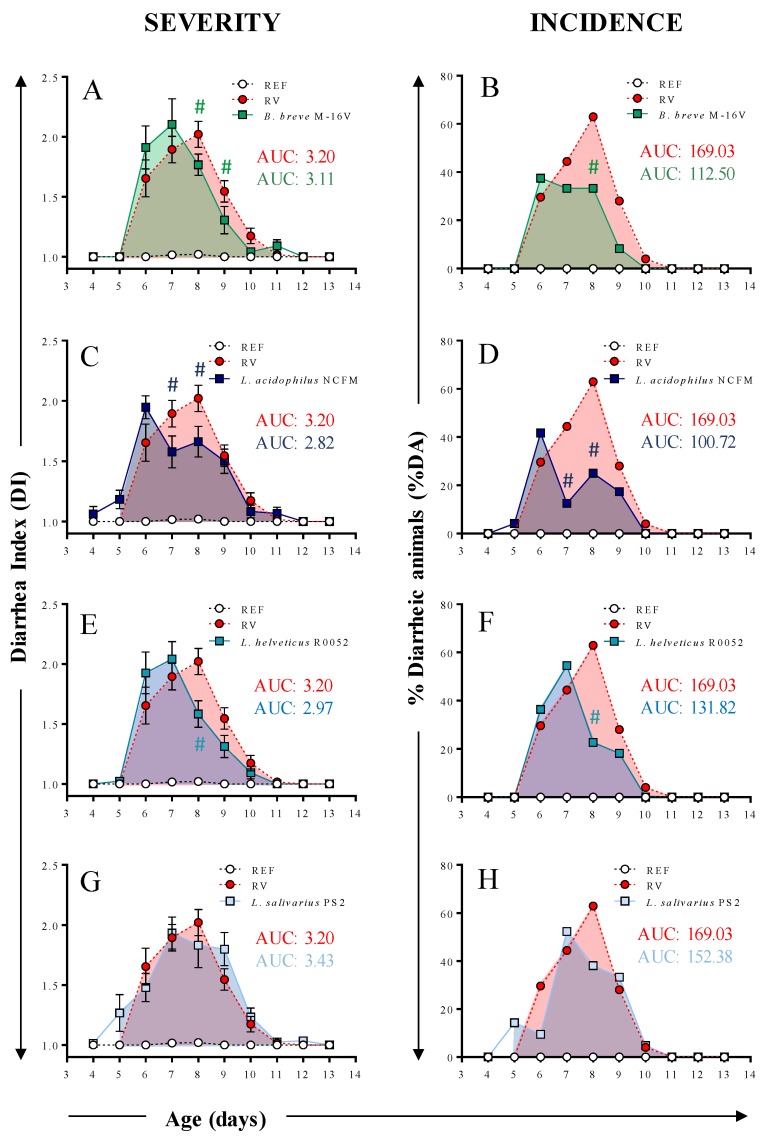
Clinical indices of diarrhea. Severity and incidence of diarrhea in animals receiving (**A**,**B**) *B. breve* M-16, (**C**,**D**) *L. acidophilus* NCFM, (**E**,**F**) *L. helveticus* R0052, and (**G**,**H**) *L. salivarius* PS2. The severity is expressed with the diarrhea index (DI) on a scale of 1 to 4. Scores of DI ≥ 2 indicate presence of diarrhea. The incidence of diarrhea is represented as the percentage of diarrheic animals (%DA), which corresponds to the percentage of animals displaying DI scores ≥ 2 in each group. The mean area under the curve (AUC) of severity and incidence, S-AUC and I-AUC, respectively, is represented with a colored shadow and its value is displayed in the right side of the graph. Results are expressed as mean ± S.E.M. (*n* = 4–21/group, depending on fecal sample availability). ^#^ *p* < 0.05 compared to the rotavirus (RV) group. REF: reference; *B*.: *Bifidobacterium*; *L*.: *Lactobacillus*.

Diarrhea was studied in terms of severity and incidence variables (Figure 2 and Table 2). RV infection resulted in a mild diarrhea in suckling rats, with DI scores around 2–3, showing the peak of severity (MSd) and incidence (MId) on day 8 and an overall prevalence of diarrhea (%DA) of 88.9%, similarly to previous studies [13,56]. The *B. breve* M-16V, *L. acidophilus* NCFM, and *L. helveticus* R0052 groups displayed clinical amelioration of severity (DI) and incidence (%DA) during the peak of diarrhea (*p* < 0.05 on day 8, Figure 2A–F). Moreover, animals receiving *B. breve* M-16V showed reduced DI on day 9 (*p* < 0.05, Figure 2A) and animals receiving *L. acidophilus* NCFM showed reduced DI and %DA on day 7 (*p* < 0.05, Figure 2C,D). The severity and incidence of the group supplemented with *L. salivarius* PS2 was similar to that of the RV group.

The mean severity-area under the curve (S-AUC) was calculated as a good indicator of the global process. Accordingly, the reduction in the DI score observed in the *B. breve* M-16V, *L. acidophilus* NCFM, and *L. helveticus* R0052 groups during the peak of diarrhea was reflected in a lower value of the S-AUC compared to the RV group (Figure 2A,C,E). Similarly, these three probiotic strains displayed lower values of the I-AUC (Figure 2B,D,F), with *L. acidophilus* NCFM being the most effective (~41% reduction), followed by *B. breve* M-16V (~34% reduction) and *L. helveticus* R0052 (~22% reduction). The S-AUC and I-AUC of the group supplemented with *L. salivarius* PS2 was comparable to the RV group.

Other variables associated to the severity, incidence and duration of diarrhea were also calculated (Table 2). The maximum severity (MS) was similar in all groups. However, the maximum severity day (MSd) was approximately half a day earlier in the animals receiving *B. breve* M-16V, *L. acidophilus* NCFM, and *L. helveticus* R0052, as compared to the RV group (*p* < 0.05). The maximum incidence (MI) was reduced by supplementations with *B. breve* M-16V (~40% reduction) and *L. acidophilus* NCFM (~34% reduction), as compared to the RV group (*p* < 0.05). Interestingly, all probiotic supplementations displayed the maximum incidence day (MId) between one day (*L. helveticu*s and *L. salivarius* strains) and two days (*B. breve* and *L. acidophilus* strains) before the RV group. The duration of the diarrhea was also modified by probiotic supplementations. In this regard, animals receiving *B. breve* M-16V and *L. acidophilus* NCFM displayed the shortest duration, with a more than 50% reduction in both the diarrhea period (DP) and the days with diarrhea (DwD), as compared to the RV group (*p* < 0.05). In contrast, the supplementation with *L. helveticus* R0052 only evidenced a shorter DP (~27% reduction), as compared to the RV group (*p* < 0.05). None of these variables were affected due to the supplementation with *L. salivarius* PS2.

Finally, *L. salivarius* PS2 did not show any statistically significant differences as compared to the RV group in any of the variables analyzed.

### 3.3. Fecal Weight and pH

**Figure 3:**
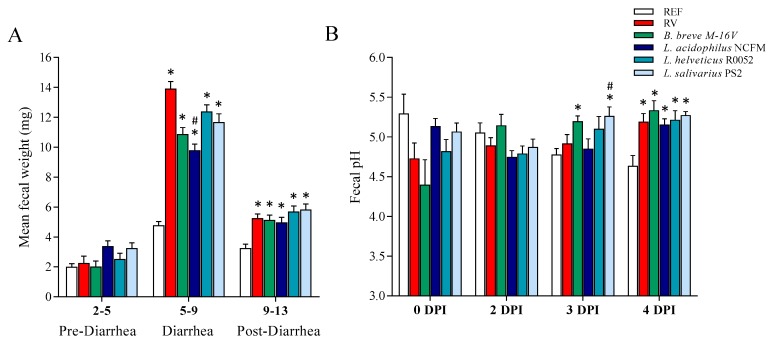
Fecal variables. (**A**) Mean fecal weight during the pre-diarrhea, diarrhea, and post-diarrhea periods. (**B**) Fecal pH before the infection (0 days post-inoculation, DPI) and after the infection (2, 3, and 4 DPI). Results are expressed as mean ± S.E.M. (*n* = 4–21/group, depending on fecal sample availability). * *p* < 0.05 compared to the reference (REF) group; ^#^ *p* < 0.05 compared to the rotavirus (RV) group. *B*.: *Bifidobacterium*; *L*.: *Lactobacillus*.

The fecal weight was measured throughout the study (Figure 3A), allowing us to characterize the diarrheic process. No differences in fecal weight were detected in the pre-diarrheic process in any of the groups. However, in the diarrhea period all groups infected with RV increased the fecal weight compared to the REF group (*p* < 0.05). All probiotic supplementations displayed a lower mean fecal weight in this period, from which only *L. acidophilus* NCFM evidenced statistical differences with the RV group (*p* < 0.05), although *B. breve* M-16V showed a tendency to reduction.

The fecal pH was measured before the infection (0 DPI) and after the infection (2, 3, and 4 DPI) (Figure 3B). No differences in fecal pH were observed at 0 and 2 DPI. Conversely, at 3 DPI the group supplemented with *B. breve* M-16V displayed a higher pH compared to REF group and the same was seen for *L. salivarius* PS2 compared to both the REF and RV groups (*p* < 0.05). Finally, all infected groups displayed a higher pH at 4 DPI compared to the REF group (*p* < 0.05), regardless of the probiotic supplementations.

### 3.4. Viral Shedding

**Figure 4:**
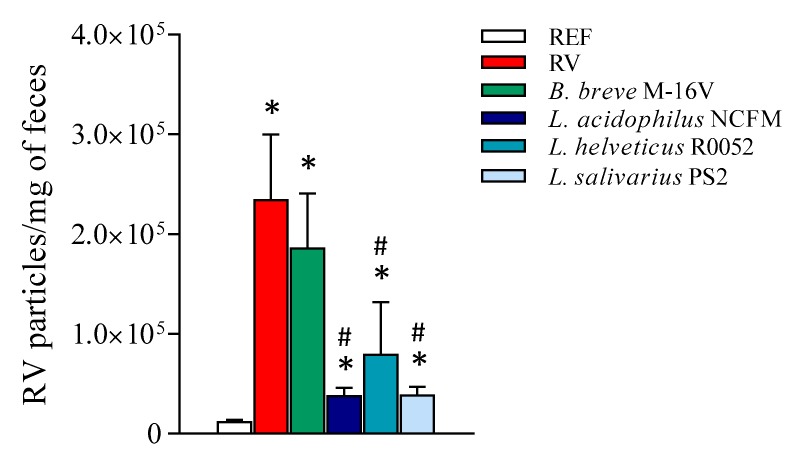
Fecal rotavirus elimination. The detection of rotavirus particles was performed in feces 1 DPI, which corresponded to the day of maximum elimination. Results are expressed as mean ± S.E.M. (*n* = 6–10/group, depending on fecal sample availability). * *p* < 0.05 compared to the reference (REF) group; ^#^ *p* < 0.05 compared to the rotavirus (RV) group. *B*.: *Bifidobacterium*; *L*.: *Lactobacillus*.

The viral shedding was determined by ELISA at 1 DPI (Figure 4), which corresponded to the day of maximum elimination of RV [13,56]. All RV-infected groups displayed higher RV elimination compared to the REF group (*p* < 0.05), which acted as the background signal (no RV infection). All supplementations with *Lactobacillus* strains displayed a more than 60% reduction in the viral detection compared to the RV group (*p* < 0.05). No differences were detected when supplementing suckling rats with the *Bifidobacterium* strain.

### 3.5. Anti-RV Humoral Response

**Figure 5:**
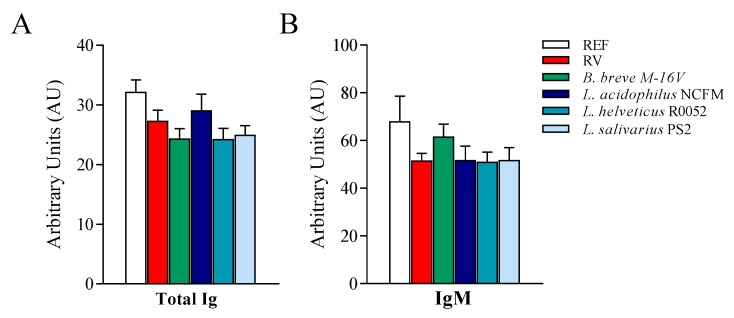
Specific anti-RV immunoglobulins (Ig). The quantification of anti-rotavirus (RV) Ig in plasma was performed by ELISA at the end of the study (day 14). The relative amount of (**A**) total and (**B**) IgM anti-RV Ig are displayed for the different experimental groups. Results are expressed as mean ± S.E.M. (*n* = 24/group) of arbitrary units (AUs). The highest dilution of the standard (pooled sera) corresponded to 1 AU. REF: reference; *B*.: *Bifidobacterium*; *L*.: *Lactobacillus*.

Once the RV infection was resolved, and coinciding with the end of the study (day 14), the determination of anti-RV specific Ig was performed (Figure 5). No differences were detected in the levels of anti-RV total Ig and IgM due to the RV infection or the probiotic supplementations.

## 4. Discussion

The role of probiotics in modulating RV diarrhea has gained interest in the past few decades. In this context, there is evidence that several *Bifidobacterium* and *Lactobacillus* strains are effective against RV gastroenteritis [7,13]. Moreover, nowadays there is a vast number of probiotics commercially available, although only a few have been tested in the context of RV. Therefore, in the present study we performed a preclinical screening of four probiotic strains in the RV SA-11 model. The rationale behind the probiotic selection was established through pediatric use or following their source of origin linking with early life (i.e., isolation from infant feces or human milk). Moreover, some of them are actually included in infant formulas [50,57,58]. The dose was established in accordance with other studies [59,60]. The results of this study show a significant protective effect against RV-induced diarrhea in animals administered daily with *B. breve* 16-MV, *L. acidophilus* NCFM, and *L. helveticus* R0052. 

RV infection did not modify the body weight of the animals in terms of absolute body weight and body weight gain. Nevertheless, we detected a slight decrease of the BMI at the end of the study in the group infected with RV without probiotic supplementation. This may suggest that probiotic intervention may prevent this low weight loss associated with RV infection. Overall, the induced diarrhea was moderate and did not have a relevant effect on the weight of the animals, which is a similar finding from previous studies in our lab using this RV SA-11 model [13,30,36,56]. 

The severity and incidence of RV diarrhea in the present study was moderate and similar to previous studies [13,30,36,56]. This model is optimal for characterizing nutritional components because it induces mild diarrhea. Stronger diarrhea models, such as those in mice and piglets [61,62], would most probably hinder the nutritional effects. Another advantage of this model is its high prevalence of ~90% in the RV group (percentage of animals with DI > 2 throughout the study).

All probiotics displayed positive effects in the clinical assessment of the RV diarrhea, with the exception of *L. salivarius* PS2 which did not modify the course of the diarrhea. *B. breve* M-16V, *L. acidophilus* NCFM, and *L. helveticus* R0052 reduced the severity of diarrhea on day 8, which corresponded with the day with the maximum severity (MSd) in the RV group. Moreover, these probiotics clearly reduced the incidence of diarrhea on the day of the maximum incidence (MId), reducing the I-AUC ~20%–40%, and thus indicating that they were effective in ameliorating RV diarrhea. Importantly, the duration of diarrhea, which is one of the outcomes analyzed in most studies, was also shortened (by 25%–60%). In previous studies in our lab we also found decreased severity, incidence, and duration of diarrhea following supplementation with *B. breve* M-16V [13,36]. Interestingly, a study by Liu et al. supplementing gnotobiotic pigs with *L. acidophilus* NCFM, the same strain used here, reported reduced duration and clinical score only when administering intermediate doses of the probiotic (10^3^–10^6^ CFU/day) [63]. Additionally, there is scientific evidence that *L. acidophilus* displays efficacy in reducing RV diarrhea duration in children [16,20,32,37,63]. However, most studies are performed with a mixture of probiotics, and the specific strain used is not detailed. Furthermore, although the severity of the disease is not usually scored directly, other indicators such as reduction of fever or reduced days of hospitalization are often reported [20,32,37]. In addition, *L. helveticus* R0052 seems to be a good candidate to ameliorate RV diarrhea. Nevertheless, there are no studies available in the context of RV, and thus, this opens up a new window for further study of this probiotic. Finally, the lack of action of *L. salivarius* PS2 in the clinical variables analyzed does not necessary imply that the strain is not effective against RV diarrhea, but rather that it did not show any effect in the conditions used here (e.g., dose, age of the animals, RV strain, length of the treatment). The probiotic strain *L. salivarius* LMG P-27027 (also referred to as *L. salivarius* PS2), has been recently isolated from human milk and has been shown in a clinical study to prevent infectious mastitis in lactating women by decreasing the mastitis incidence rates [64]. Furthermore, the authors demonstrated that when mastitis occurred, the milk bacterial counts in the probiotic group were significantly lower than those obtained in the placebo group. These results might suggest that *L. salivarius* LMG P-27027 (*L. salivarius* PS2) is effective in alleviating bacterial infection-related symptoms and not those related to viral infection.

The fecal weight is also a good objective indicator of the severity of diarrhea. In the present study we detected a three-fold increase in the fecal weight of the RV group compared to the REF group. All probiotic supplementations displayed a lower mean fecal weight during the diarrhea period, especially in the group supplemented with *L. acidophilus* NCFM, which reduced the mean fecal weight relative to the RV group by 30%. Accordingly, clinical studies in children reported a reduction in the number of stools per day after the supplementation with *L. acidophilus* [20,32]. Although in the present study we found a tendency to reduce the fecal weight in the *B. breve* M-16V group, previous studies in our group using this probiotic in the RV SA-11 model showed a clear reduction [13,36]. 

The fecal pH can also be an indicator of the diarrhea process because the intestinal pH is reduced as a consequence of the malabsorption of carbohydrates and electrolyte imbalance [65,66]. Conversely to past studies in our laboratory, we did not detect a drop in fecal pH due to RV infection in the peak of the diarrhea period [67]. Instead, we detected an increase of the fecal pH in the end of the diarrhea period in all groups infected with RV, independently of probiotic supplementation. Another study in our laboratory using a double infection RV model also displayed an increase of pH at 1 DPI in the primary infection [36]. Further studies should be conducted for its use in the assessment of the diarrhea process.

When RV arrives in the small intestine it infects epithelial cells and starts the replication process. Then, a high viral load is found in feces as a reflection of the elimination of the initial virus inoculum and the new virions produced. Herein, RV elimination in feces at 1 DPI, coinciding with the day of maximum elimination [13,56], was notably reduced in the groups supplemented with the *Lactobacillus* strains, but not *Bifidobacterium*. Accordingly, previous studies using *B. breve* M-16V did not show any reduction of viral shedding [13,36]. However, other *Bifidobacterium* strains, such as *B. bifidum*, have shown activity in the reduction of viral elimination in infants [68] and in mice [69]. On the other hand, herein the reduction of viral shedding exerted by *Lactobacillus* is in line with other studies, which found that *L. rhamnosus* GG was able to reduce RV elimination in gnotobiotic piglets infected with human RV [14] and in children [59]. In vitro studies have reported that *Bifidobacteria* and *Lactobacillus* are able to inhibit RV infection [2,37], for example by interfering in the adhesion step. In this regard, it may be possible that glycans or other molecules found on the surface of the bacteria interact with the virus, therefore blocking the infection, similar to oligosaccharides present in breastmilk [70,71] and infant formula [13].

Finally, the specific anti-RV antibody response was not modified by the administration of probiotics, most probably because the humoral response on day 14 of life is still immature or is hindered by the Ig transferred through breastmilk. Indeed, no differences were observed between the non-infected and infected groups.

## 5. Conclusions

The present study provides insight into the potential of supplementation with specific probiotic strains in early life to prevent RV diarrhea. Particularly, this study highlights the differential efficacy of four bacterial strains, thus reinforcing the idea that probiotics have strain-specific effects [72,73]. *L. acidophilus* NCFM is a highly effective probiotic in the present experimental design, in addition to *B. breve* M-16V and *L. helveticus* R0052. Previous data in our lab also are in line with the finding that *B. breve* M-16V may prevent RV diarrhea [13,36]. A thorough study should be performed with *L. acidophilus* NCFM and *L. helveticus* R0052, which have shown promising effects in the present model. Moreover, although one of the means by which these probiotics confer protection might be the reduction of viral shedding, more studies should be performed in order to decipher the exact mechanisms of action involved.

## Figures and Tables

**Table 1:** Growth-associated measurements at the end of the study (day 14).

	REF	RV	*B. breve* M-16V	*L. acidophilus* NCFM	*L. helveticus* R0052	*L. salivarius* PS2
**Morphometrics**						
BMI (g/cm^2^)	0.31 ± 0.02	0.29 ± 0.01 *	0.30 ± 0.01	0.30 ± 0.00	0.30 ± 0.00	0.32 ± 0.01 ^#^
Body/tail length ratio	2.12 ± 0.02	2.11 ± 0.04	2.05 ± 0.03	2.09 ± 0.02	2.03 ± 0.02	2.07 ± 0.03
Lee Index (g^0.33^/cm × 10^3^)	330.86 ± 2.90	323.97 ± 2.92	325.50 ± 2.51	326.77 ± 2.03	329.64 ± 2.44	335.44 ± 3.79 ^#^

Results are expressed as mean ± S.E.M. (*n* = 24/group). * *p* < 0.05 compared to the reference (REF) group. *^#^p* < 0.05 compared to the rotavirus (RV) group. BMI: body mass index; *B*.: *Bifidobacterium*; *L*.: *Lactobacillus*.

**Table 2:** Variables describing severity, incidence and duration of diarrhea.

	RV	*B. breve* M-16V	*L. acidophilus* NCFM	*L. helveticus* R0052	*L. salivarius* PS2
**Severity**					
MS	2.20 ± 0.07	2.14 ± 0.13	2.06 ± 0.10	2.13 ± 0.12	2.23 ± 0.14
MSd	7.40 ± 0.18	6.87 ± 0.21 ^#^	6.82 ± 0.21 ^#^	6.72 ± 0.23 ^#^	7.14 ± 0.26
**Incidence**					
MI	62.96	37.50 ^#^	41.66 ^#^	54.54	52.38
MId	8	6	6	7	7
**Duration**					
DDB	7.00 ± 0.18	6.84 ± 0.21	6.58 ± 0.23	6.62 ± 0.18	6.87 ± 0.31
DDE	8.04 ± 0.19	7.29 ± 0.23	7.27 ± 0.30	7.63 ± 0.24	8.06 ± 0.32
DP	0.93 ± 0.22	0.36 ± 0.12 ^#^	0.36 ± 0.17 ^#^	0.68 ± 0.23 ^#^	0.91 ± 0.28
DwD	1.67 ± 0.21	1.14 ± 0.17 ^#^	0.86 ± 0.15 ^#^	1.35 ± 0.26	1.52 ± 0.29

Results are expressed as mean ± S.E.M. (*n* = 12–24/group depending on fecal sample availability), ^#^ *p* < 0.05 compared to the rotavirus (RV) group. MS: maximum severity; MSd: maximum severity day; MI: maximum incidence; MId: maximum incidence day; DDB: day of diarrhea beginning; DDE: day of diarrhea ending; DP: diarrhea period; DwD: days with diarrhea. REF: reference; *B*.: *Bifidobacterium*; *L*.: *Lactobacillus*.
